# Integrated Boost vs. Sequential Scheme in Volumetric Modulated Arc Therapy (VMAT) for Trimodal Bladder Cancer Therapy: A Comparative Study

**DOI:** 10.7759/cureus.99438

**Published:** 2025-12-17

**Authors:** Reyzane El Mjabber, Rim Alami, Fatimaezzahra Aouzah, Wissam Bezzari, Zineb Dahbi, Fadila Kouhen, Nabil Ismaili, Sanaa El Majjaoui, Asmaa Naim

**Affiliations:** 1 Radiation Oncology, Mohammed VI University of Sciences and Health (UM6SS), Casablanca, MAR; 2 Radiation Oncology, International University Hospital Cheikh Khalifa, Casablanca, MAR; 3 Radiotherapy, Mohammed VI University of Sciences and Health (UM6SS), Casablanca, MAR; 4 Medical Oncology, Mohammed VI University of Sciences and Health (UM6SS), Casablanca, MAR; 5 Medical Oncology, International University Hospital Cheikh Khalifa, Casablanca, MAR

**Keywords:** bladder preservation, boost radiotherapy, muscle-invasive bladder cancer (mibc), trimodal therapy, volumetric‐modulated arc therapy (vmat)

## Abstract

Background

Trimodal therapy (TMT), combining transurethral resection of the bladder tumor, radiotherapy, and concurrent chemotherapy, is an established bladder-preserving approach for muscle-invasive bladder cancer (MIBC). Advances in radiotherapy, such as volumetric modulated arc therapy (VMAT), have enabled the use of different dose delivery schemes, namely simultaneous integrated boost (SIB) and sequential boost (SEQ) techniques. This study aimed to compare dosimetric outcomes, organ-at-risk sparing, and treatment tolerance between these two VMAT schemes.

Methods

Fourteen patients with MIBC treated with TMT between December 2016 and December 2021 were retrospectively analyzed. Seven patients received SIB-VMAT, and seven received SEQ-VMAT. Treatment plans were generated using Eclipse^®^ software (Varian Medical Systems, Palo Alto, CA, United States), and dose-volume parameters were evaluated for the rectum, small bowel, and femoral heads. Tumor response and toxicities were assessed according to CTCAE v6.0 criteria. Statistical analyses were performed using IBM SPSS Statistics for Windows, Version 25.0 (Released 2017; IBM Corp., Armonk, NY, USA).

Results

Target coverage and conformity were comparable between SIB and SEQ plans (V98%: 95.7% vs. 96.8%; V2%: 102.2% vs. 101.9%). Rectal and femoral head doses were similar across both schemes, whereas the small bowel received higher V15Gy and V45Gy in the SIB group (863.6 mL and 130.6 mL vs. 517.3 mL and 71.6 mL, respectively). Acute toxicities were predominantly urinary (Grade 1-2 cystitis) and occurred in 21.4% of patients. The complete response rate was slightly higher in the SIB group (18%) compared with the SEQ group (17%), although the difference was not statistically significant.

Conclusions

Both SIB and SEQ-VMAT schemes provide effective and well-tolerated treatment options for bladder preservation in MIBC. SIB offers a shorter overall treatment duration and marginally improved target conformity, while SEQ may better spare the small bowel at lower dose levels. The choice between these approaches can be individualized according to patient anatomy and institutional expertise.

## Introduction

Trimodal therapy (TMT), combining maximal transurethral resection of the bladder tumor (TURBT) followed by concurrent chemoradiotherapy, has emerged as a robust and widely accepted bladder-sparing strategy for patients with muscle-invasive bladder cancer (MIBC) [1]. For appropriately selected patients, bladder preservation offers oncologic outcomes comparable to radical cystectomy while maintaining superior quality of life, particularly regarding urinary, sexual, and social functioning [1,2]. Radiotherapy is a cornerstone of this multimodal approach, serving not only to provide local tumor control but also to eradicate microscopic disease that may remain after TURBT.

Over the past decade, major technological advances have redefined radiotherapy planning and delivery for bladder cancer. The integration of image guidance, adaptive strategies, and highly conformal techniques such as volumetric modulated arc therapy (VMAT) has markedly improved the therapeutic ratio. VMAT allows for enhanced dose modulation, tighter conformity around complex bladder targets, and improved sparing of critical organs at risk (OARs), including the rectum, bowel, and femoral heads [3]. These advantages are particularly relevant for bladder irradiation, where target volumes are large, mobile, and anatomically variable.

Within VMAT-based bladder radiotherapy, two principal dose-delivery strategies are currently used. The sequential boost (SEQ) approach administers an initial dose to a broader pelvic or whole-bladder volume, followed by a second, higher-dose phase targeted at the bladder [4]. In contrast, the simultaneous integrated boost (SIB) approach delivers differential dose levels to multiple target volumes within the same treatment fraction, allowing dose escalation to the tumor while shortening overall treatment time. Although both strategies are widely implemented in clinical practice, evidence directly comparing their dosimetric performance, acute and late toxicity profiles, and clinical outcomes remains limited.

Given the growing interest in bladder preservation and the increased availability of advanced radiotherapy techniques, a clearer understanding of the relative benefits and limitations of SEQ vs. SIB is essential to optimize treatment protocols.

The primary objective of this study was to compare the dosimetric performance of SIB-VMAT and SEQ-VMAT boost techniques, specifically in terms of target coverage and OAR sparing. Secondary objectives were to evaluate acute treatment-related toxicity and early tumor response within a trimodal bladder-preservation strategy for MIBC.

## Materials and methods

Study design and setting

This retrospective study was conducted over a five-year period, from December 14, 2016, to September 22, 2025, and included all patients with histologically confirmed bladder cancer (squamous cell carcinoma and urothelial carcinoma) who received radiotherapy using the RapidArc technique at the Department of Radiation Oncology, Cheikh Khalifa International University Hospital.

Study population

The study included 14 newly diagnosed patients with histologically confirmed non-metastatic bladder cancer treated at the Department of Radiation Oncology, Cheikh Khalifa International University Hospital. Patients were assigned to one of two treatment groups: Group A, receiving SIB using VMAT (SIB-VMAT), and Group B, receiving SEQ with VMAT (SEQ-VMAT). The allocation of patients to the SIB or SEQ group was not randomized. No predefined standardized criteria were used for decision-making; instead, the choice of technique was at the discretion of the treating physician based on clinical considerations and individual patient characteristics. Relevant clinical and treatment data were retrieved from patients’ electronic medical records in DxCare, and follow-up assessments were conducted during oncology and radiotherapy consultations.

Inclusion Criteria

In accordance with Radiation Therapy Oncology Group (RTOG) guidelines [4], eligible patients were adults (≥18 years) with histologically confirmed bladder cancer, including muscle-invasive disease (T2-T3) and selected non-muscle-invasive cases (Ta) who had undergone prior focal therapy without response and declined surgery, all without distant metastases (M0) [5]. Patients were required to have a functional bladder without significant scarring or anatomical abnormalities and to provide written informed consent after being fully informed of the risks and benefits of treatment. Additional inclusion criteria were the ability to undergo complete tumor resection via TURBT, adequate performance status (ECOG 0-2) [6], and sufficient renal, hepatic, and hematologic function to tolerate concomitant chemotherapy. Patients with uncontrolled severe urinary infections or a history of prior pelvic radiotherapy were excluded. The indication for radiotherapy was established for all patients through multidisciplinary tumor board discussion, and all participants were able to comply with regular follow-up visits for assessment of treatment response and monitoring of treatment-related toxicities.

Exclusion Criteria

Exclusion criteria were established in accordance with RTOG guidelines to ensure methodological consistency and rigor [4]. Patients with distant metastases (M1) [5] or those unable to provide informed consent were excluded. Additional exclusions included patients with a nonfunctional bladder, significant scarring, or anatomical abnormalities that would preclude safe delivery of radiotherapy, as well as those with poor performance status (ECOG >2) or insufficient renal, hepatic, or hematologic function [6]. Patients with uncontrolled severe urinary infections or a history of prior pelvic radiotherapy were also excluded. Furthermore, individuals unable to adhere to regular follow-up for assessment of treatment response and monitoring of toxicities, or those with incomplete medical records or lost to follow-up during treatment, were not included in the study.

Treatment planning and delivery

CT Simulation

CT simulation was performed as the initial step after confirming patient eligibility, with the aim of acquiring high-resolution 3D anatomical data for precise treatment planning. Patients were positioned supine on the CT simulation table and immobilized using ankle stocks and knee bolsters to ensure reproducibility throughout the treatment course. Standardized preparation protocols were applied, including fasting, rectal emptying (using mild laxatives or enemas when indicated to minimize rectal variability), and maintaining a comfortably full bladder by consuming 200 mL of water approximately 30 minutes before imaging to improve targeting accuracy [4].

Target Volumes Delineation 

Target volumes and OARs were meticulously delineated on the simulation CT scans in accordance with RTOG guidelines [4]. Delineation was carried out using Eclipse^®^ software (Varian Medical Systems, Palo Alto, CA, United States) by a team of four experienced radiation oncologists. To ensure consistency, accuracy, and adherence to protocol, all contours were cross-checked within the team, and any discrepancies were resolved through consensus.

Radiotherapy was planned following RTOG guidelines using a two-phase approach. CTV1 (clinical target volume) included the entire bladder. For tumors involving the bladder neck, trigone, or multifocal disease (CIS), additional structures were included: the prostatic urethra and prostate in males and the pelvic urethra and bladder sphincter in females. Elective pelvic lymph nodes included obturator, internal and external iliac, presacral, and common iliac nodes. The CTV boost encompassed the entire bladder to deliver a higher dose to areas at greatest risk. Planning target volumes (PTVs) were defined by adding margins to the CTVs to account for setup uncertainties and internal organ motion: PTV T1 = CTV1 + 1.5 cm, PTV T2 = CTV boost + 1.5 cm, and PTV N = CTV nodal + 0.7 cm [4].

Dose Delivery

For SEQ-VMAT patients, the first phase encompassed PTV T1, delivering 45-46 Gy in 1.8-2 Gy daily fractions to the bladder and elective pelvic nodes. The second phase delivered a boost to PTV T2 and any involved nodes (N+), escalating the total dose to 64-66 Gy while preserving bladder function. For SIB-VMAT patients, PTV nodes and the bladder received 45-50 Gy in 1.8-2 Gy per fraction, while PTV boost to the bladder and involved nodes simultaneously received 64-66 Gy in 2-2.2 Gy per fraction [4].

Treatment Planning and Dosimetric Assessment

Treatment planning was conducted on the same Treatment Planning System by three departmental medical physicists, following institutional protocols and quality assurance procedures. These included verification of target volumes and OARs, review of dose distributions and dose-volume histograms, compliance with OAR constraints, machine-specific checks of linear accelerator output and beam constancy, and final plan validation by both the physicist and the radiation oncologist prior to initiation of treatment.

Treatment Delivery

Radiotherapy was delivered using two linear accelerators (Clinac IX and TrueBeam, Varian Medical Systems) with daily fractions administered five days per week according to the prescribed dose and fractionation schedules. Image-guided radiotherapy was performed prior to each treatment session. Cone-beam CT was acquired during the first three fractions and then weekly, while daily kilovoltage (kV-kV) imaging was used for all remaining fractions to verify alignment and maintain treatment precision throughout the course.

Assessment of Toxicities

Treatment-related adverse events were systematically recorded and graded using the Common Terminology Criteria for Adverse Events (CTCAE), version 5.0 [7]. Acute toxicities were assessed in all patients and compared between the two treatment groups.

Assessment of Tumor Response

Therapeutic response was evaluated endoscopically. For SEQ-VMAT patients, the first cystoscopic assessment was performed after delivery of 46 Gy. In the event of documented disease progression at this stage, patients were referred for salvage surgery; otherwise, treatment continued according to the initially planned approach. For SIB-VMAT patients, therapeutic response was assessed six to eight weeks after completion of radiotherapy by cystoscopy. Patients achieving a favorable response were followed with cystoscopic and clinical surveillance, whereas those exhibiting disease progression were referred for salvage surgery.

Statistical analysis

Statistical analyses were conducted using IBM SPSS Statistics for Windows, Version 25.0 (Released 2017; IBM Corp., Armonk, NY, USA). Continuous variables were summarized using means, SDs, and ranges, while categorical variables were expressed as frequencies and percentages. Comparisons between groups were performed using appropriate parametric or non-parametric tests based on data distribution. A p-value <0.05 was considered statistically significant.

## Results

Patient characteristics

Fourteen patients with MIBC were treated with TMT, consisting of maximal TURB followed by concomitant chemoradiotherapy, according to European Society for Medical Oncology (ESMO) eligibility criteria (localized, solitary, completely resected tumor; no hydronephrosis; no prostatic urethra involvement; no concomitant carcinoma in situ). The cohort included 12 men and two women, with a median age of 72.14 years. Tumor stages were Ta in two (14.28%) patients and T3 in four (28.57%) patients. Nodal stages were N0 in 10 (71.4%) patients, N1 in two (14.28%) patients, and N2 in two (14.28%) patients.

Seven patients (50%) received an integrated boost, with total doses of 64-64.5 Gy in 30 fractions (2.13-2.15 Gy/fraction). The remaining seven patients (50%) received a sequential scheme with 66 Gy in 33 fractions (2 Gy/fraction). Targets included pelvic lymph nodes in all 14 (100%) patients, encompassing obturator, presacral, internal iliac, external iliac, and common iliac lymph nodes. A boost to lymph nodes was delivered in four (28.57%) patients (Table 1).

**Table 1:** Patient characteristics, dosimetry, PTV coverage, and toxicities in the comparative study of SIB vs. sequential VMAT for bladder cancer PTV, planning target volume; SIB, simultaneous integrated boost; VMAT, volumetric modulated arc therapy

Parameter	Subcategory/value	N (%)
Age	Mean	72.14 (62-91)
Sex	Male/female	12/2
Classification	T2N0M0	6 (42.85%)
	TaN0M0	2 (14.28%)
	T2N2M0	2 (14.28%)
	T3N0M0	2 (14.28%)
	T3N1M0	2 (14.28%)
Scheme	SIB	7 (50%)
	Sequential scheme	7 (50%)
Total dose	66 Gy	7 (50%)
	64.5 Gy	6 (42.85%)
	64 Gy	1 (7.14%)
Dose per fraction	2 Gy	7 (50%)
	2.15 Gy	6 (42.85%)
	2.13 Gy	1 (7.14%)
Rectum V60Gy	0%	7 (50%)
	2%	1 (7.14%)
	36%	1 (7.14%)
	23.70%	1 (7.14%)
	1.33%	1 (7.14%)
	0.84%	1 (7.14%)
	9.18%	1 (7.14%)
	15.91%	1 (7.14%)
Small bowel V15Gy	534 cc	1 (7.14%)
	1595 cc	1 (7.14%)
	634 cc	2 (14.28%)
	1718 cc	1 (7.14%)
	524 cc	1 (7.14%)
	254 cc	1 (7.14%)
	546 cc	1 (7.14%)
	372 cc	1 (7.14%)
	462 cc	1 (7.14%)
	523 cc	2 (14.28%)
Small bowel V45Gy	25.7 cc	1 (7.14%)
	347 cc	1 (7.14%)
	81 cc	1 (7.14%)
	34 cc	1 (7.14%)
	125 cc	1 (7.14%)
	105 cc	2 (14.28%)
	51 cc	1 (7.14%)
	83 cc	1 (7.14%)
	19.2 cc	1 (7.14%)
	39.8 cc	2 (14.28%)
Right femoral head V50Gy	0%	13 (92.85%)
	4.81%	1 (7.14%)
Left femoral head V50Gy	0%	13 (92.85%)
	1.30%	1 (7.14%)
PTV V2%	66.5 Gy	1 (7.14%)
	67.9 Gy	1 (7.14%)
	67 Gy	1 (7.14%)
	66.9 Gy	1 (7.14%)
	68.4 Gy	1 (7.14%)
	67 Gy	1 (7.14%)
	66 Gy	1 (7.14%)
	65 Gy	1 (7.14%)
	67.88 Gy	1 (7.14%)
	65.45 Gy	1 (7.14%)
	67 Gy	1 (7.14%)
	65.6 Gy	1 (7.14%)
	65.2 Gy	1 (7.14%)
	61.2 Gy	1 (7.14%)
PTV V98%	61.3 Gy	1 (7.14%)
	60.5 Gy	1 (7.14%)
	64.6 Gy	1 (7.14%)
	64.8 Gy	1 (7.14%)
	63 Gy	1 (7.14%)
	64.8 Gy	1 (7.14%)
	61.2 Gy	1 (7.14%)
	62.9 Gy	1 (7.14%)
	63.27 Gy	1 (7.14%)
	62.3 Gy	1 (7.14%)
	64.7 Gy	1 (7.14%)
	62.5 Gy	1 (7.14%)
	62.34 Gy	1 (7.14%)
	61.2 Gy	1 (7.14%)
Toxicity	Cystitis Grade 1	1 (7.14%)
	Cystitis Grade 2	2 (14.28%)
Evolution	Local recurrence	4 (28.57%)
	Distant recurrence	5 (35.71%)
	Free survival	3 years

Dosimetric parameters

Rectum

**Figure 1:**
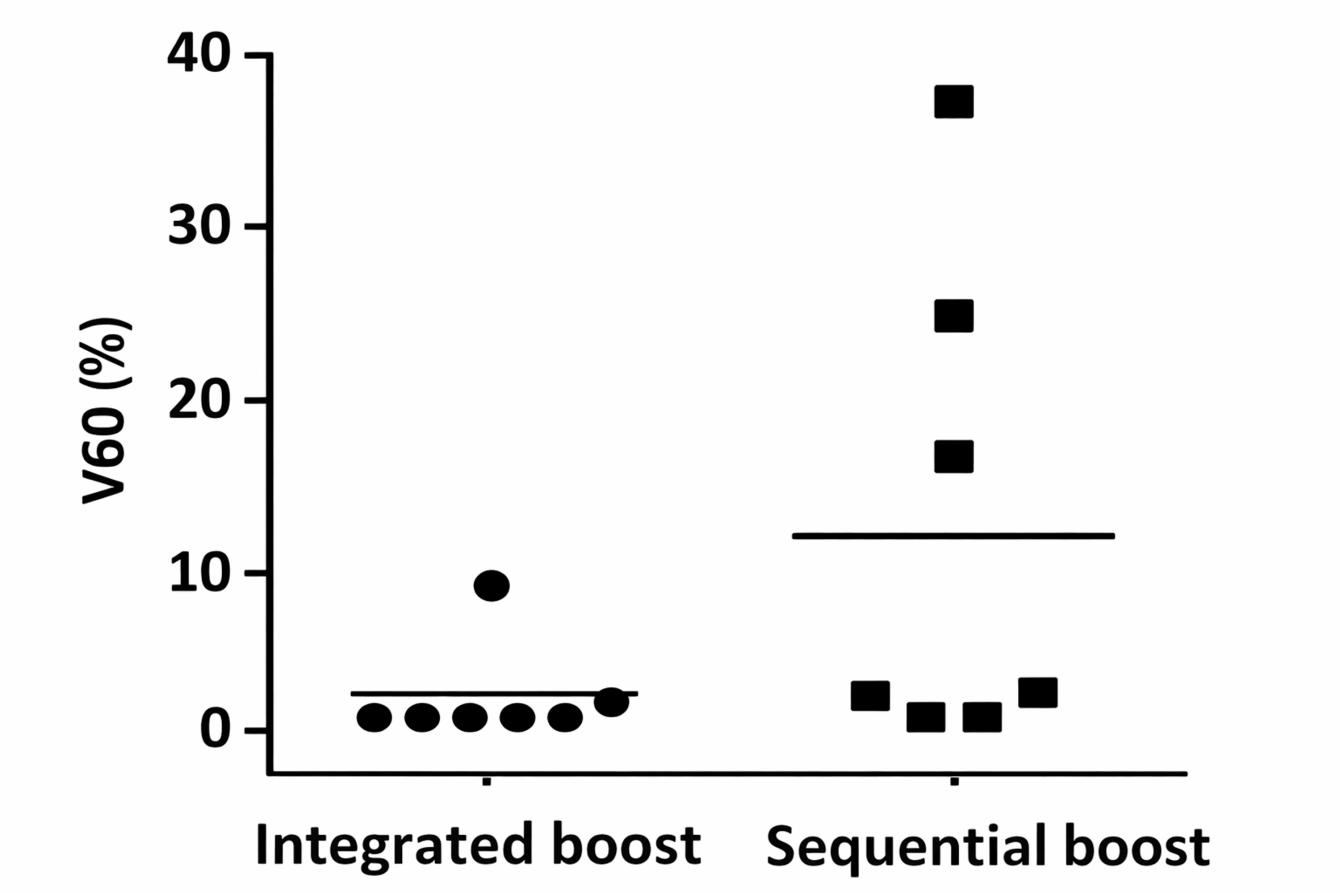
Rectal V60 (%) in the integrated boost vs. sequential boost groups (p = 0.0781)

Mean V60Gy was 1.43% for the integrated boost and 11.27% for the sequential scheme, indicating comparable rectal sparing (p = 0.0781) (Figure 1).

Small Bowel

**Figure 2:**
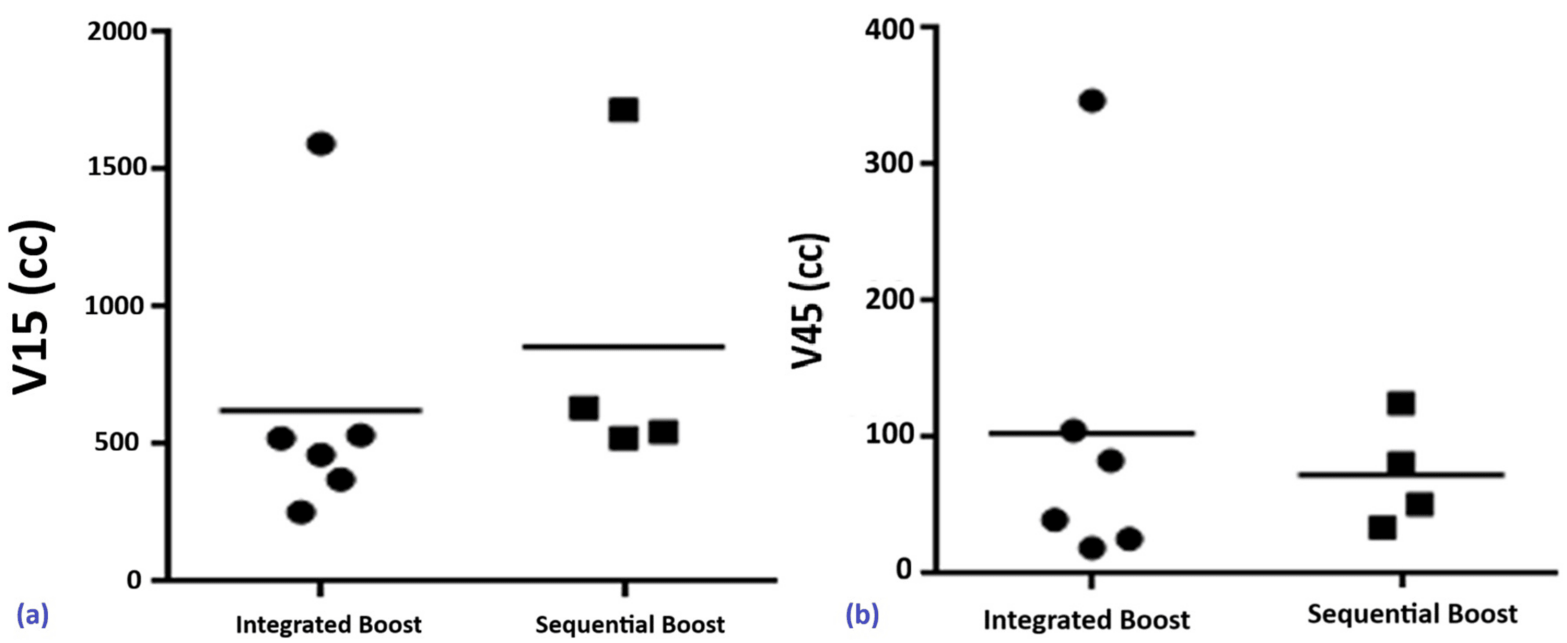
Comparative small bowel doses: (a) V15Gy (p = 0.114) and (b) V45Gy (p = 0.895) for the two regimens

Mean doses of V15Gy and V45Gy were higher in the integrated boost group (V15Gy: 863.6 mL; V45Gy: 130.6 mL) compared to the sequential scheme (V15Gy: 517.3 mL; V45Gy: 71.6 mL). However, the differences were not statistically significant (V15Gy: p = 0.114; V45Gy: p = 0.895) (Figure 2a, 2b).

Femoral Heads

**Figure 3:**
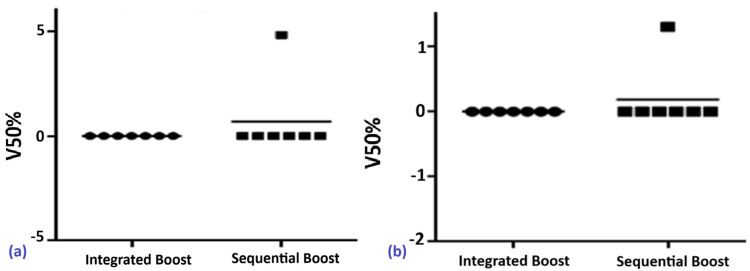
Distribution of V50Gy to the right (a) and left (b) femoral heads and necks in patients treated with SIB vs. SEQ SEQ, sequential boost; SIB, simultaneous integrated boost

In the integrated boost group, all seven patients (100%) had V50Gy = 0%. In the sequential group, two patients (14.3%) had V50Gy of 4.81% (right) and 1.3% (left). No statistically significant differences were observed between the two schemes (p ≈ 1) (Figure 3a, 3b).

**Figure 4:**
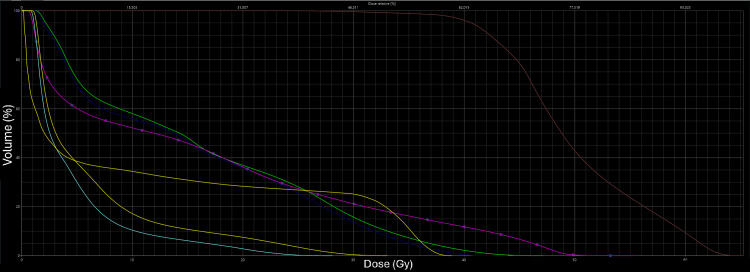
Dose-volume histogram for OAR in the SIB VMAT plan OAR, organ at risk; SIB, simultaneous integrated boost; VMAT, volumetric modulated arc therapy

**Figure 5:**
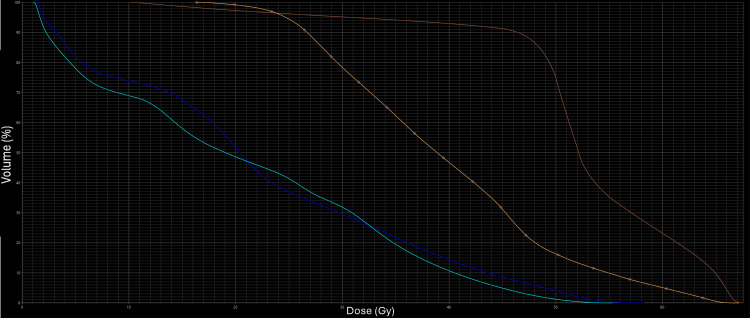
Dose-volume histogram for OAR in the sequential scheme VMAT plan OAR, organ at risk; VMAT, volumetric modulated arc therapy

These results indicate that both approaches are feasible, with the sequential scheme potentially offering advantages in sparing the small bowel and femoral heads, which may reduce toxicity (Figure 4, Figure 5).

PTV coverage and treatment response

**Figure 6:**
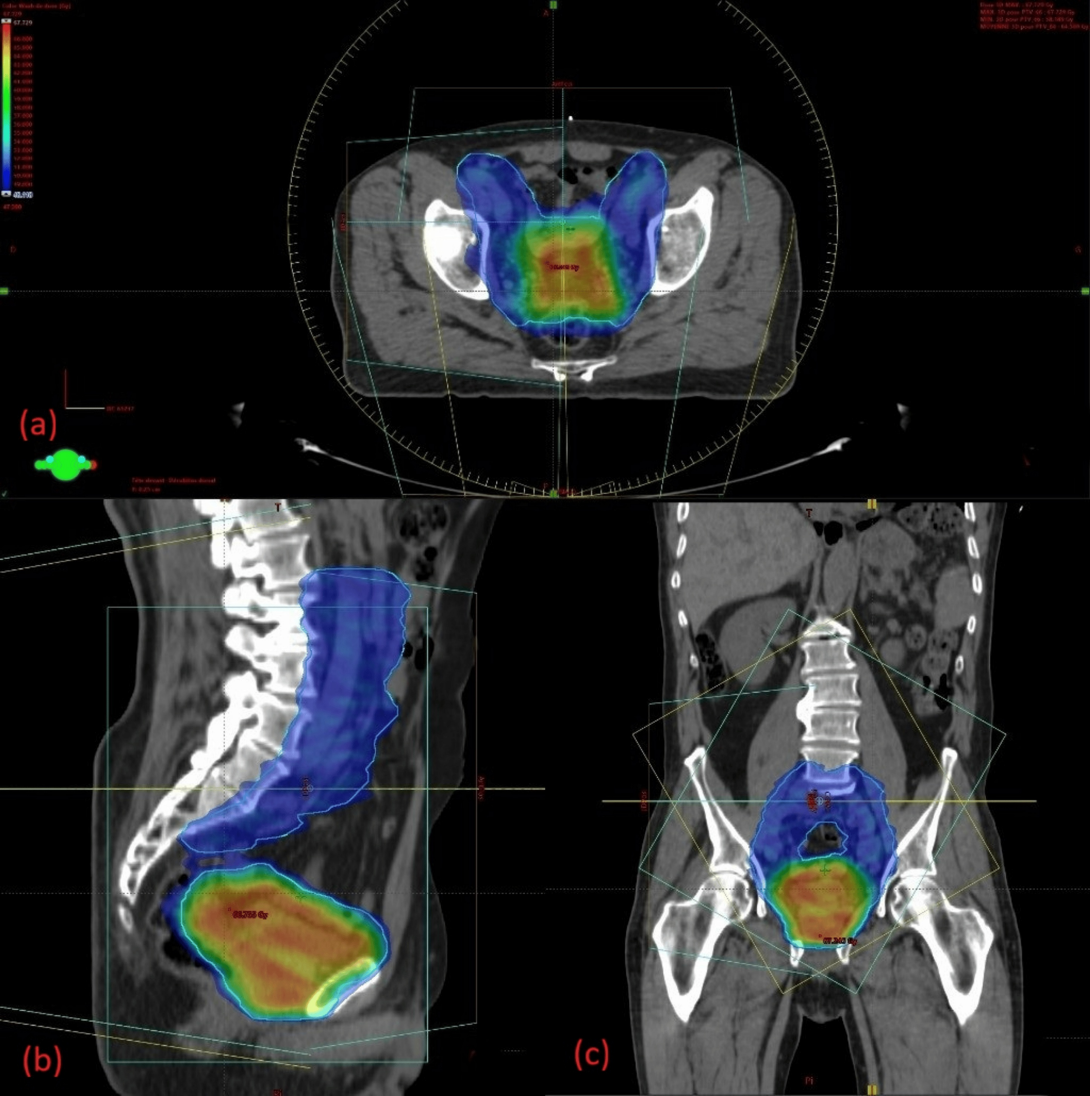
Dose distribution for the integrated boost to the bladder tumor Axial (a), sagittal (b), and coronal (c) views illustrating the VMAT plan delivering an integrated boost dose of 64.5 Gy to the PTV (blue contour). The color wash shows the dose gradient, with higher doses concentrated in the boost region while ensuring adequate sparing of surrounding OARs. OAR, organ at risk; PTV, planning target volume; VMAT, volumetric modulated arc therapy

**Figure 7:**
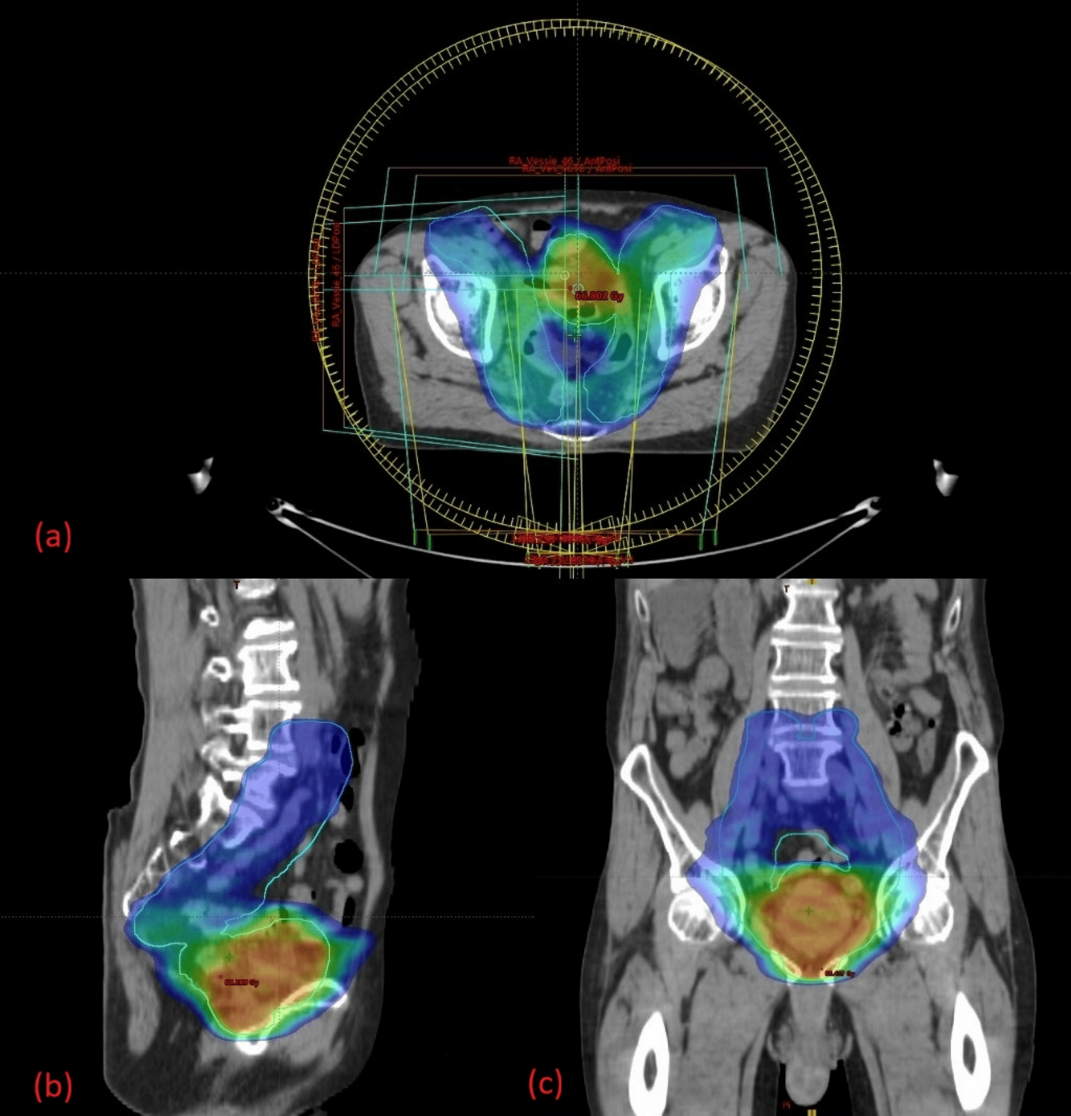
Dose distribution for the sequential boost to the bladder tumor Axial (a), sagittal (b), and coronal (c) views illustrating the VMAT plan delivering a sequential boost dose of 66 Gy to the PTV. The color wash depicts the high-dose region concentrated on the boost volume, with appropriate sparing of surrounding pelvic OARs. OAR, organ at risk; PTV, planning target volume; VMAT, volumetric modulated arc therapy

For the integrated boost regimen, the PTV received a mean V2% dose of 65.94 Gy (102.2% of the prescribed dose) and a mean V98% dose of 61.77 Gy (95.7%). In the sequential scheme, the mean V2% dose was 67.24 Gy (101.9%) and the mean V98% dose was 63.93 Gy (96.9%) (Figure 6a-6c, Figure 7a-7c).

During treatment, all 14 patients (100%) underwent re-evaluation cystoscopy. Overall, five patients (35.7%) achieved a complete response: three patients (21.42%) in the sequential group and two patients (14.28%) in the integrated boost group. Six patients (42.85%) showed a partial response with residual tumor, and three patients (21.42%) experienced tumor progression. No patients underwent salvage cystectomy, as all opted for a bladder-preserving approach.

Tolerance and toxicities

Treatment was well tolerated in 11 (78.5%) patients. Toxicities were observed in three (21.4%) patients and were predominantly urinary. One patient (7.14%) experienced pelvic pain, acute urinary retention, and cystitis, while two patients (14.28%) developed grade 2 cystitis with hematuria. All three patients (21.4%)who experienced toxicities had received the integrated boost regimen. All adverse events were successfully managed with symptomatic treatment, with no long-term sequelae.

Outcomes and survival

At median follow-up, four (28.57%) patients achieved favorable outcomes, nine (64.28%) experienced tumor progression, and one (7.14%) died.

## Discussion

In this study, we evaluated 14 patients with MIBC treated with TMT, comparing two radiotherapy schemes: SIB-VMAT and sequential VMAT. The primary aim was to assess dosimetric differences, OAR protection, target coverage, treatment tolerance, and tumor response, providing insights into the feasibility and safety of these approaches. Both schemes were feasible and generally well-tolerated, with SIB offering slight advantages in treatment duration and PTV coverage. The choice between SIB and sequential schemes should be individualized, considering patient anatomy, tumor volume, and bladder preservation goals. SIB allows delivery of higher doses per fraction in a shorter overall treatment time, which may enhance tumor response without increasing toxicity, making it a promising approach in centers with experienced staff and advanced planning capabilities [4].

Dosimetric analysis revealed that the sequential scheme better spared the small bowel at low-dose volumes, whereas SIB achieved slightly improved PTV coverage. Specifically, the sequential regimen resulted in V15Gy 517.3 mL and V45Gy 71.6 mL, compared to 863.6 mL and 130.6 mL for SIB, indicating a clinically relevant reduction in low-dose exposure to this parallel organ, consistent with findings reported by Roeske et al. [8] and Nuyttens et al. [9]. Rectal constraints were respected in both schemes (V60Gy 6.25% for sequential vs. 6.45% for SIB), as were femoral head constraints (V50Gy: 0% SIB, 4.81% right and 1.3% left sequential), in agreement with prior studies [10]. These results illustrate the trade-off between slightly better PTV coverage with SIB and improved low-dose OAR sparing with sequential therapy, reflecting fundamental radiobiological principles for parallel vs. serial organs [11].

Treatment tolerance was generally favorable. All patients receiving sequential VMAT completed therapy without significant adverse events, while 57.1% of patients treated with SIB tolerated therapy uneventfully. Observed toxicities were exclusively urinary (Grade 1-2 cystitis, hematuria, pelvic pain, and acute urinary retention), and all were successfully managed symptomatically, aligning with literature highlighting bladder-specific toxicity in TMT [10,11].

Post-treatment cystoscopy showed a complete response rate of 36.7% (17% sequential, 18.3% SIB), partial response in 38.3%, and tumor progression in 25%, with no patients undergoing salvage cystectomy, confirming the feasibility of bladder preservation after visually complete TURBT. This aligns with pooled analyses by Mak et al. [4] and long-term data from Rödel et al. [12], demonstrating that complete TURBT is a key predictor of response. Although our complete response rate is lower than that reported by Kaufman et al. [13] (81%) and Kachnic et al. [14] (66%), it surpasses the overall survival reported in James et al. [15] (48%) and approaches the 71-75% survival described by Mitin et al. [16], highlighting variability due to patient selection, tumor stage, and TURBT completeness.

Analysis of T-stages revealed a range from Ta to T4, with most trials including T2-T4a disease. In prior RTOG studies (85-12, 88-02, 97-06) [16-18] and the series by Rödel et al. [12], tumor stage was not predictive of complete response, consistent with our findings. Visual inspection of isodose curves demonstrated no hot spots in the PTV, with V98% 95.7% (SIB) vs. 96.9% (sequential) and V2% 102.2% vs. 101.9%, adhering to ICRU 83 recommendations and ensuring homogeneity [4,19-21].

The accuracy of dosimetric results was influenced by planner dependency, differences in contouring among radiation oncologists, physicist experience, and patient morphology. Despite this, constraints for PTV and OARs were respected, except for minor deviations in SIB plans where slight increases in low-dose exposure to the small bowel were accepted to improve PTV coverage. This underscores the importance of individualized planning and consideration of organ architecture, with parallel organs tolerating higher doses in small volumes and serial organs requiring stringent dose limits [8].

Overall, our findings support that both SIB and sequential VMAT are safe and effective for TMT in MIBC. In this preliminary analysis, SIB appeared to provide slightly improved PTV coverage and shorter treatment duration, whereas sequential VMAT better limited low-dose exposure to parallel OARs. These observations align with current principles emphasizing maximal TURBT, judicious patient selection, and adherence to dosimetric constraints.

This study has several limitations. The small sample size (14 patients) and its retrospective, single-center design limit generalizability. Variability in treatment planning and organ delineation among multiple clinicians may have influenced dosimetric outcomes. Differences in dose, fractionation, and target volumes between SIB and sequential schemes, along with the lack of randomization, may confound comparisons. Additionally, follow-up duration was limited, and no formal assessment of patient-reported outcomes was performed, restricting evaluation of long-term toxicity, bladder function, and predictors of treatment response.

Future prospective studies are warranted to optimize treatment strategies, incorporate adaptive radiotherapy, and evaluate predictive biomarkers for treatment response, thereby improving the therapeutic ratio and bladder preservation outcomes.

## Conclusions

Based on our experience with 14 patients, integrated boost radiotherapy (IBRT) significantly reduces overall treatment duration while allowing higher doses per fraction to be delivered to the target volume. Both the SIB and sequential irradiation schemes provided comparable target coverage and maintained adherence to dosimetric constraints, with a suggestion of marginally improved coverage for SIB. Treatment tolerance and tumor response were similar between the two approaches, supporting the possibility of individualizing the choice of regimen based on patient anatomy, tumor characteristics, and bladder preservation goals.

Nevertheless, the small sample size limits the generalizability of our findings. Further studies with larger cohorts are warranted to confirm the efficacy of IBRT and optimize treatment parameters for different patient populations. Overall, this study provides valuable insights into the potential benefits of IBRT in bladder cancer management and encourages further research to improve patient outcomes.
